# Bilateral Giant Full Thickness Macular Holes: An Infrequent Manifestation of Alport Syndrome

**DOI:** 10.18502/jovr.v18i3.13781

**Published:** 2023-07-28

**Authors:** Saeed Karimi, Niloofar Mohammad Bagheri Rafsanjani

**Affiliations:** ^1^Ophthalmic Research Center, Research Institute for Ophthalmology and Vision Science, Shahid Beheshti University of Medical Science, Tehran, Iran; ^2^Department of Ophthalmology, Torfeh Medical Center, Shahid Beheshti University of Medical Sciences, Tehran, Iran; ^3^Clinical Research Development Unit of Torfeh Medical Center, Shahid Beheshti University of Medical Sciences, Tehran, Iran

**Keywords:** Alport Syndrome, Macular Hole, Pars Plana Vitrectomy

## Abstract

**Purpose:**

To report a case of Alport syndrome presenting with bilateral giant full-thickness macular holes, hypertensive chorioretinopathy, and exudative retinal detachment.

**Case Report:**

A 20 year-old man, a known case of Alport syndrome on hemodialysis, was referred to our clinic with bilateral vision loss initiated about 10 years prior to presentation, which exacerbated in the month prior to our visit. Bilateral large full-thickness macular holes, hypertensive chorioretinopathy, and exudative retinal detachment were detected in fundus examination. The patient had previous genetic counseling confirming the diagnosis of Alport syndrome. During follow-up, macular holes were covered with a thick epiretinal membrane and visual acuity decreased progressively in two weeks. Pars plana vitrectomy was performed in the right eye. Two weeks following surgery, the macular hole was closed and visual acuity improved significantly.

**Conclusion:**

Bilateral giant full-thickness macular holes are uncommon presentations of Alport syndrome. The retinal findings may be caused by an inefficient type IV collagen presenting in the Bruch's membrane and in the internal limiting membrane. Pars plana vitrectomy can be considered to repair macular holes in these patients.

##  INTRODUCTION 

Alport syndrome is a condition with an inheritance pattern caused by basement membrane dysfunction. The X-linked patterns can impair renal, ocular, and auditory systems. Mutation in the COL IV A V gene on chromosome X is the most common pattern of Alport syndrome.^[1,3]^ The COL IV A III and COL IV A IV genes mutations are other causes of Alport syndrome.^[1,4]^ Collagen type IV is one of the most important components of basement membranes, which is present in the normal anterior and posterior lens capsules, ciliary body, Bowman's layer of the cornea, Descemet's membrane, internal-limiting membrane (ILM), retinal pigment epithelium (RPE), and basal lamina of the Bruch's membrane. Thinning of these membranes may be the principal pathophysiological mechanism of Alport syndrome. ^[5]^

The significant ocular manifestations of Alport syndrome are retinopathies and anterior lenticonus. Anterior lenticonus is caused by the thinning of the anterior lens capsule and the presence of small defects in the anterior lens capsule, a decrease in the number of anterior lens epithelial cells, and bulging of the anterior cortex through the thinned capsule. In the X-linked inheritance pattern, anterior lenticonus occurs in up to 70% of affected adult males so every man with anterior lenticonus should be screened for Alport. This condition is uncommon in adult females.

Detection of an oil droplet reflex during retinoscopy may be the only ocular sign revealed in patients with Alport syndrome created by a thin cornea with ectasia, which is generally greatest at the apex of the cone.^[16]^ Central and peripheral retinopathies are present in 50–80% of affected adult males and up to 25% of affected adult females. The Alport syndrome retinopathy presents as whitish–yellow peri-macular dots and flecks.^[6]^ Temporal macular thinning in inner retinal layers, choroidal thinning, and mid-peripheral retinoschisis are the most common abnormalities in X-linked Alport syndrome detected by optical coherence tomography.^[8,15]^ Electroretinogram (ERG) and electrooculogram (EOG) diagnostic tests also reveal bilateral abnormalities.^[15]^ In this case report, we present a rare ocular manifestation of Alport syndrome.

**Figure 1:**
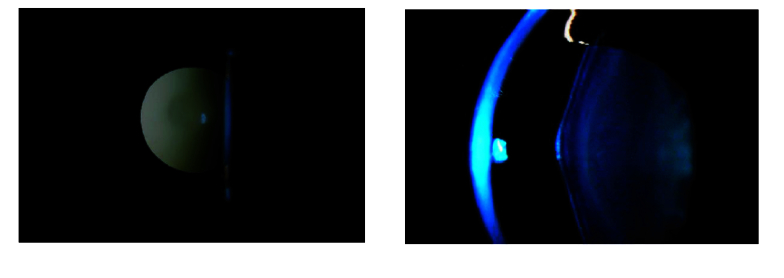
Slit photography showing anterior lenticonus and retroillumination technique photography showing anterior lenticonus in the right eye.

**Figure 2:**
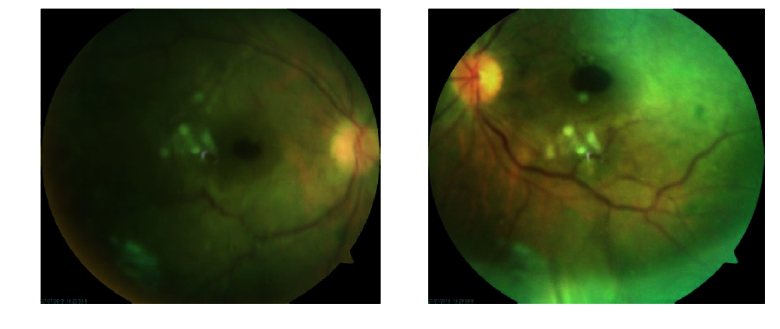
Fundus photography at first examination showing bilateral full thickness macular holes.

**Figure 3:**
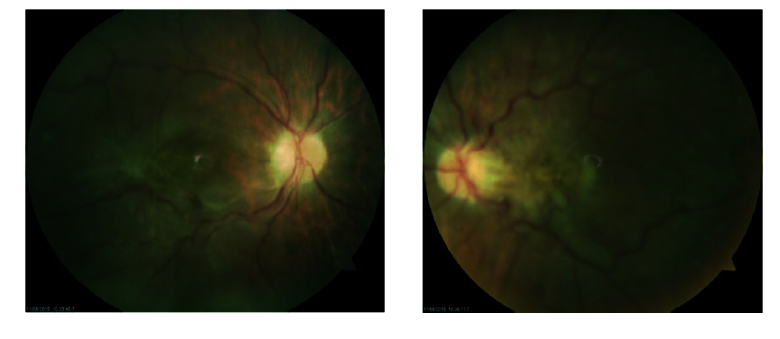
Fundus photography after two weeks' follow-up showing ERM covering the macular holes.

**Figure 4:**
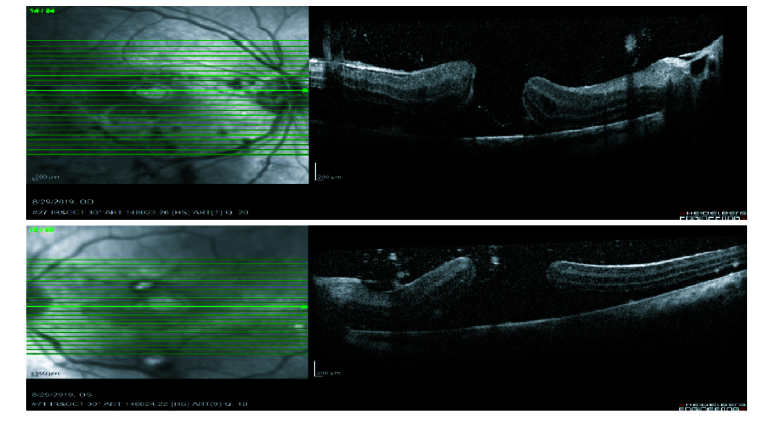
Macular OCT at first examination showing bilateral full thickness giant macular holes.

**Figure 5:**
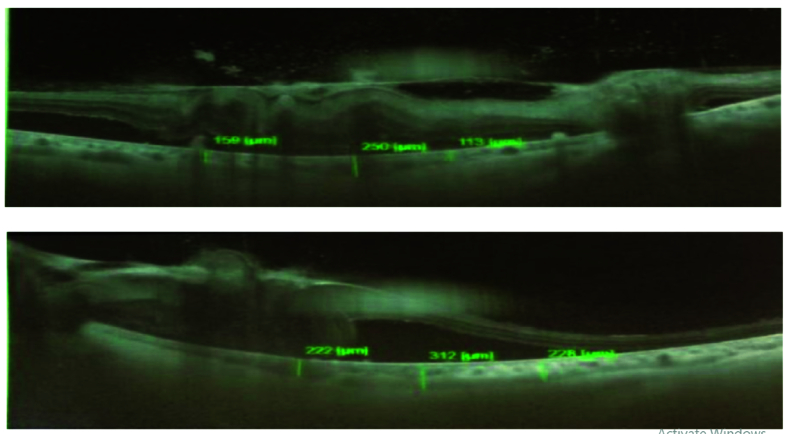
Macular OCT after two weeks' follow-up showing thick ERM covering macular holes.

**Figure 6:**
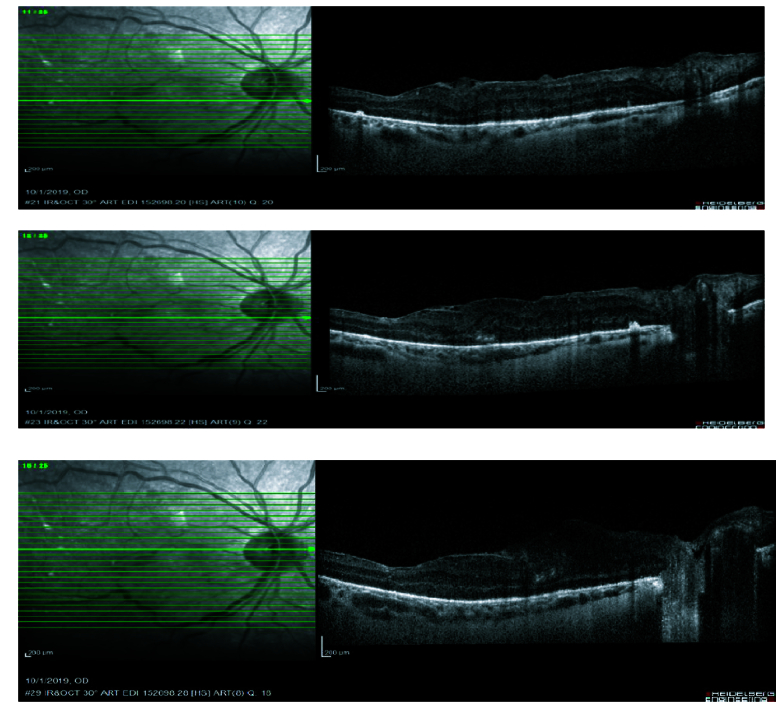
Macular OCT two weeks after vitrectomy showing closed macular hole.

##  CASE REPORT

A 20-year-old man who was a known case of Alport syndrome was referred with bilateral progressive painless vision loss. Visual symptoms, which started 10 years prior to presentation, were exacerbated in the month before our visit. His medical history included sensorineural hearing loss, renal failure, and systemic arterial hypertension. He was currently on hemodialysis and on the waiting list for a kidney transplant.

Best-corrected visual acuity (BCVA) was counting fingers at 2 m in the right eye (OD) and at 4 m in the left eye (OS) at the first visit. Slit-lamp examination revealed anterior lenticonus in crystalline lenses. Intraocular pressure was normal in both eyes. Fundoscopy showed bilateral full-thickness macular holes, hypertensive chorioretinopathy, and exudative retinal detachment in both eyes. Spectral domain optical coherence tomography (SD-OCT) showed bilateral full-thickness macular holes and vitreous concentrations of microparticles which increased after retinal detachment. It seemed that vitreous cells were photoreceptors that were shed in the vitreous cavity after retinal detachment [Figure 5].

In the second week, macular holes were covered with a thick epiretinal membrane and the patient's visual acuity decreased significantly. Pars plana vitrectomy, peeling of epiretinal membrane and internal limiting membrane (ILM) followed by fluid-gas exchange with 20% hexafluoride (SF6) were performed for the right eye. Two weeks following the surgery, the macular hole was closed and visual acuity improved to 20/100. The left eye was scheduled for a similar surgery but the patient missed the follow-up appointments.

##  DISCUSSION

In this case report, we present a case of Alport syndrome with bilateral full-thickness macular holes. Type IV collagen disorder is one of the main causes of Alport syndrome which results in retinal manifestations such as fleck retinopathy and macular thinning.^[7,8]^ Full thickness macular hole formation is an infrequent complication of Alport syndrome.

Savige et al^[7]^ explained abnormal retinal features of Alport syndrome through the use of OCT. They attributed the distribution of the dot-and-fleck retinopathy and the thinning of the temporal macula to ILM/retinal nerve fiber layer hyperreflectivity revealed on the OCT and suggested that ILM and Bruch's membrane thinning might be the initial causes of temporal macular thinning and macular hole formation. The difference between Alport syndrome–associated macular holes and idiopathic macular holes may be determined by the factors such as the age of onset and the vitreoretinal interface status.^[5,7]^

Miller et al suggested that macular hole surgery might be beneficial for selected patients with Alport syndrome,^[13]^ which was consistent with our findings. Improvement of visual acuity occurred in our patient two weeks after the surgery. On the contrary, Raimundo et al proposed that conservative treatment might be more beneficial for bilateral giant macular holes in Alport syndrome, due to unsuccessful results following vitreoretinal surgery.^[14]^

The decision for choosing surgery as a viable treatment option should be based on the signs revealed by a meticulous ocular examination such as the amount of prior damage to the photoreceptor layer. There are some reports in the literature declaring that the size of macular hole in patients with Alport syndrome may increase during the follow-up. Furthermore, macular ischemic damage and enlargement of the macular hole may be observed in patients with chronic hypertensive nephropathy.^[12]^

In summary, bilateral giant full-thickness macular holes are uncommon presentations of Alport syndrome. The retinal findings may be caused by an inefficient type IV collagen presenting in the Bruch's membrane and in the ILM. Pars plana vitrectomy, epiretinal membrane and ILM peeling, and intravitreal injection of SF6 were performed for the right eye in our patient. Two weeks following the surgery, the macular hole was closed and visual acuity improved significantly. Pars plana vitrectomy can be considered as an option for macular hole repair in these patients; however, the outcome is not conclusive as it depends on the medical history and the level of retinal damage that may exist.

##  Financial Support and Sponsorship

None.

##  Conflicts of Interest

None.
